# Novel Applications of OnabotulinumtoxinA in Lower Urinary Tract Dysfunction

**DOI:** 10.3390/toxins10070260

**Published:** 2018-06-26

**Authors:** Jia-Fong Jhang, Hann-Chorng Kuo

**Affiliations:** Department of Urology, Buddhist Tzu Chi General Hospital, and Tzu Chi University, Hualien 970, Taiwan; alur1984@hotmail.com

**Keywords:** botulinum toxin, clinical trial, human, urodynamics

## Abstract

OnabotulinumtoxinA (BoNT-A) was first used to treat neurogenic lower urinary tract dysfunction (LUTD) 30 years ago. Recently, application of BoNT-A in LUTD have become more common since the approval of intravesical BoNT-A injection for patients with both overactive bladders (OAB) and neurogenic detrusor overactivity (NDO) by regulatory agencies in many countries. Although unlicensed, BoNT-A has been recommended to treat patients with interstitial cystitis/bladder pain syndrome (IC/BPS) under different guidelines. BoNT-A delivery with liposome-encapsulation and gelation hydrogel intravesical instillation provided a potentially less invasive and more convenient form of application for patients with OAB or IC/BPS. BoNT-A injections into the urethral sphincter for spinal cord injury patients with detrusor-sphincter dyssynergia have been used for a long time. New evidence revealed that it could also be applied to patients with non-neurogenic dysfunctional voiding. Previous studies and meta-analyses suggest that BoNT-A injections for patients with benign prostate hyperplasia do not have a better therapeutic effect than placebo. However, new randomized and placebo-controlled trials revealed intraprostatic BoNT-A injection is superior to placebo in specific patients. A recent trial also showed intraprostatic BoNT-A injection could significantly reduce pain in patients with chronic prostatitis. Both careful selection of patients and prudent use of urodynamic evaluation results to confirm diagnoses are essential for successful outcomes of BoNT-A treatment for LUTD.

## 1. Introduction

Lower urinary tract dysfunction (LUTD) is a condition presented by patients suffering from LUTD linked to one or more structures and/or functions of the lower urinary tract [1]. LUTD is common in both men and women, and the incidence and prevalence increase as people age [2]. A recent large cross-sectional study in China revealed 61.1% of women and 61.2% of men reported LUTD [2]. In general, the pathophysiology of LUTD could be classified into bladder or bladder outlet dysfunction, and treatments of LUTD should focus on etiology. However, not all LUTDs could be treated effectively, even if the etiology is definite. Treatment of functional LUTD, such as interstitial cystitis/bladder pain syndrome (IC/BPS), overactive bladder (OAB), and dysfunctional voiding (DV), remains a challenge to the urologist.

Botulinum toxin (BoNT) is a potent poisonous neurotoxin, which is produced by the bacterium *Clostridium botulinum* and related species [3]. Ingestion of BoNT-poisoned food causes intoxication by inhibiting the release of the neurotransmitter acetylcholine from nerve fibers, thereby inhibiting muscle contractions, which was first described in the early 17th century [4]. BoNT was first isolated in 1895, and now could be classified antigenically and serologically into eight distinguishable exotoxins (A, B, C1, C2, D, E, F, and G) [5]. In 1981, Scott first used onabotulinumtoxinA (BoNT-A) by injecting it into human eye muscles to correct strabismus successfully [6]. Since then, BoNT-A has been widely used to treat many neuropathic pain syndromes and dystonic diseases. In LUTD, the first application of BoNT-A targeted the urethral sphincter. Dykstra used transperineal or cystoscopic injection of BoNT-A into the urethral sphincter in patients with spinal cord injury (SCI) and detrusor-sphincter dyssynergia (DSD) in 1988 [7]. Currently, BoNT-A has been widely used in different kinds of LUTDs, especially in diseases that could not be easily treated with oral medications. In the American Urology Association (AUA) guidelines, BoNT-A injection into the urinary bladder is now a standard treatment for patients with refractory OAB and IC/BPS [8,9]. Newly published clinical trials also revealed novel applications of BoNT-A in different LUTDs and exhibited promising results. The aim of the current article is to review important new applications of BoNT-A in LUTDs and the associated evidence supporting its use.

## 2. Mechanisms of BoNT-A in LUTDs

BoNT-A, a potent neurotoxic protein, is well known for its ability to inhibit the release of the neurotransmitter acetylcholine from presynaptic efferent nerves at neuromuscular junctions [5]. BoNT-A consists of a 50-kDa light chain and a 100-kDa heavy endocytosis [10]. Subsequently, the light chain and heavy chain separate in the endosomal vesicle [11,12]. The light chain is the biologically active moiety of BoNT-A. The light chain of BoNT-A cleaves synaptosome-associated protein 25 in the presynaptic nerve terminal, and inhibits the release of acetylcholine by disrupting the fusion of vesicles with the neuron’s cell membrane, finally causing flaccid paralysis of muscles [13,14]. Traditionally, the effects of BoNT-A in treating LUTDs, such as OAB and DSD, were believed to be attributed to the inhibition of detrusor or urethral sphincter contractions. Recently, evidence also revealed BoNT-A injection into the bladder also could regulate sensory nerve function by blocking the release of various noxious neurotransmitters, including adenosine triphosphate, calcitonin gene-related peptide, calcitonin gene-related peptide, and substance P [15,16]. Modulation of sensory nerve function might be the mechanism of BoNT-A in some sensory problems predominantly LUTD, such as IC/BPS. In addition, studies showed an anti-inflammatory effect for BoNT-A. Immunohistochemical evidence revealed decreased tryptase expression in the bladder after BoNT-A injections, which suggests a reduction in active mast cells in bladders of IC/BPS patients [17].

## 3. Intravesical BoNT-A Injection in OAB and Neurogenic Detrusor Overactivity

OAB is a clinical syndrome, which is characterized by urinary urgency, usually accompanied by frequency and nocturia, with or without urgency, urinary incontinence, in the absence of urinary tract infection (UTI), or other obvious pathology [18]. According to a recent large cross-sectional study in Asia, the prevalence of OAB was 20.8% overall (men 19.5%, women 22.1%) and increased significantly with age [19]. Treatments of OAB are usually started with behavioral therapy and then oral medications such as antimuscarinics or beta-agonists [8]. Although oral medications might be effective, a large-scale study showed that 46.2% of OAB patients discontinued antimuscarinics and stated the reason for treatment discontinuation was “did not work as expected” [20]. In patients with neurogenic detrusor overactivity (NDO) due to SCI, oral antimuscarinics have been reported to increase bladder capacity and decrease intravesical pressure [21]. However, the effect of antimuscarinics is usually poor in NDO patients with severe urgency and incontinence symptoms. In a large series of NDO patients with urinary incontinence, only 32% of patients could become continent after using oxybutynin and trospium [22]. Thus, treatment for patients with OAB and NDO, who were refractory to oral medications, is a daily common challenging problem in the urology clinic.

Intravesical BoNT-A injection for treating patients with SCI and NDO have been reported since 2000 [23]. Schurch first injected 200 to 300 units (U) of BoNT-A into the detrusor muscle of NDO patients [23]. At six weeks of follow-up, complete continence was observed in 17 of 19 (89.4%) cases in which anticholinergic medication was markedly decreased or withdrawn. Evidence from basic and clinical researchers revealed BoNT-A injection could block acetylcholine release from efferent nerves ending by cleaving Synaptosomal-associated protein 25, thereby temporarily inhibiting detrusor muscle contraction and improve bladder storage symptoms [24]. Further investigation also revealed that BoNT-A injection could also inhibit both noradrenaline and adenosine triphosphate release, which have a powerful influence on bladder sensation [25,26]. After years of work by many researchers and clinicians, intravesical BoNT-A injection has provided evidence demonstrating its utility as standard therapy in patients with both OAB and NDO [8,27]. The application of BoNT-A in OAB and NDO has also been approved by regulatory agencies in most countries.

Patients with NDO and OAB who respond to BoNT-A usually need repeat injections every 6 to 12 months [28]. The long-term efficacy of repeat BoNT-A injections was doubtful before, but recent long-term follow-up studies (>5 years) revealed that BoNT-A could decrease urinary incontinence rate and improve quality of life in patients with both NDO and OAB [29,30]. However, treatment compliance might be not satisfactory. Rahnama’i reported that only 25% of patients continued treatment during the six years of follow-up [29]. Most of these patients could not tolerate voiding urinary tract symptoms, urine retention, or urethral catheterization [29].

## 4. Intravesical Liposome-Encapsulated BoNT-A Instillation in OAB

In our prospective pilot randomized controlled study, liposome-encapsulated BoNT-A (Lipotoxin) bladder installation was used to treat patients with OAB [31]. At one month after treatment, the change in urinary frequency and urgency significantly improved in the Lipotoxin group but not in the normal saline instillation group. More importantly, no adverse event such as post-voiding residual volume (PVR), urinary retention, or UTI significantly increased or was reported by patients during the follow-up period. Bladder instillation of Lipotoxin in patients with OAB seems to be an effective treatment without significant adverse effects, but the long-term efficacy still needs to be proved in the future. Although the use of Lipotoxin for treating OAB patients is promising, it has not been used in patients with NDO until now. The therapeutic effect of Lipotoxin in NDO might be not adequate in this case.

Management is usually difficult in some patients who are characterized by urinary urgency, incontinence with incomplete bladder emptying, and with a urodynamic diagnosis of detrusor hyperactivity with impaired contractile function (DHIC) [32]. Recently, we reported our experience with suburothelial injection of 100 U of BoNT-A in patients with DHIC [33]. At six months of follow-up, the subjective urgency symptom scores improved significantly, but urgency episodes did not significantly improve in 21 patients. Acute urinary retention developed in 7 (33.3%) and UTI was noted in eight patients with DHIC (38.1%). The incidence of adverse effects of BoNT-A injection in patients with DHIC was relatively higher than that in patients with OAB. We concluded that the efficacy of intravesical BoNT-A injection for DHIC patients was limited and short-term. Physicians should inform patients of both potential benefits and risks of BoNT-A injection for the treatment of DHIC. A comprehensive urodynamic study before decision-making is recommended to rule out the coexistence of LUTD such as bladder neck dysfunction.

## 5. Intravesical BoNT-A Injection in Interstitial Cystitis/Bladder Pain Syndrome

IC/BPS is a clinical syndrome and includes a large group of patients who are defined by the AUA as having “an unpleasant sensation (pain, pressure, discomfort) perceived to be related to the urinary bladder, associated with LUTD of more than six weeks duration, in the absence of infection or other identifiable causes” [9]. The estimated prevalence of IC/BPS among adult females in the US ranges from 2.7% to 6.53% [34]. Until now, the etiology of IC/BPS is still uncertain, and its management is both frustrating and difficult [35]. Smith et al. first treated female IC/BPS patients with intravesical 100 to 200 U BoNT-A injection plus cystoscopic hydrodistention at the same time [36]. We also conducted a prospective, randomized, double-blind clinical trial to investigate the clinical efficacy of BoNT-A intravesical injection in patients with IC/BPS [37]. Our results showed a significantly greater reduction in pain and increase in cystometric bladder capacity in the BoNT-A group compared with the normal saline injection group at eight weeks of follow-up. Experimental studies conducted in both animals and humans provided laboratory evidence to support BoNT-A injections in the IC/BPS. BoNT-A injections into the bladder have been shown to block the release of noxious neurotransmitters including calcitonin, calcitonin gene-related peptide, glutamate, adenosine triphosphate, and substance P from neurons [38]. Now, intravesical BoNT-A injection is the fourth-line standard treatment in the AUA treatment guideline of IC/BPS [9].

A recent network meta-analysis compared BoNT-A injection with different intravesical therapy for IC/BPS, including bacillus Calmette-Guerin, resiniferatoxin, lidocaine, chondroitin sulfate, oxybutynin, and pentosan polysulfate [39]. The results indicate that in patients with IC/BPS BoNT-A injection has the highest probability of being the best therapy according to the global response assessment, and significantly improves bladder capacity. Recently, we conducted a double-blind, randomized trial to investigate the efficacy of intravesical Lipotoxin instillation in patients with IC/BPS [40]. Patients who received Lipotoxin therapy demonstrated a statistically significant decrease in O’Leary-Sant symptom scores and on the visual analog scale for pain. However, there was no significant difference in improvement between the Lipotoxin and normal saline instillation group. Nevertheless, no significant adverse effect developed in either group, the efficacy of Lipotxoin instillation in IC/BPS might be masked by the placebo effect, and further study is necessary to validate the actual effect. Rappaport et al. recently used TC-3 gel, a novel reverse-thermal gelation hydrogel to deliver BoNT-A into IC/BPS bladders [41]. A single intravesical instillation of 200 U of BoNT-A mixed with 40 mL TC-3 gel could significantly reduce both pain and O’Leary-Sant symptom scores at 12 weeks of follow-up. Preliminary results of instillation of a TC-3 gel-BoNT-A mixture are promising, but further prospective and randomized trials are necessary to prove its efficacy.

## 6. Urethral Sphincter BoNT-A Injection in Detrusor-Sphincter Dyssynergia

DSD is characterized by involuntary contractions of the external urethral sphincter during a detrusor contraction, which is caused by central nervous system injury between the pontine micturition center and the sacral spinal cord [42]. Patients with SCI and DSD usually suffer from incomplete bladder emptying and DO. Application of BoNT-A in DSD started as early as 1988 [7]. At that time, it was believed that the effect of BoNT-A could block acetylcholine release from presynaptic vesicles at the neuromuscular junction into the urethral sphincter [43]. However, clinical evidence to support its efficacy remains limited and randomized placebo-controlled trial data was limited until now. Our prospective study showed that 100 U of BoNT-A injection into the urethral sphincter could significantly decrease voiding detrusor pressure and increase maximum flow rate [44]. However, some patients might complain of an increase in incontinence grade and were dissatisfied with the BoNT-A injection. A recent prospective trial enrolled 59 SCI patients with both NDO and DSD. All these patients received both 200 U intravesical and 100 U urethral sphincter injections of BoNT-A at the same time [45]. Patients could experience a significant reduction of detrusor voiding pressure, urinary incontinence episode, and increased voiding volume at 12 weeks of follow-up. Twenty-five patients (42.4%) even reported complete dryness at follow-up. Patients with DSD may become incontinent after urethral sphincter BoNT-A injection, and it might adversely affect the quality of life in these patients. Simultaneously, BoNT-A injections in the detrusor and urethral sphincters are a reasonable treatment for SCI patients with both NDO and DSD. Using urodynamic study results to evaluate both urethral and bladder function in these patients and presenting a thorough explanation of all possible adverse effects as well as expectations is key to increase patient satisfaction.

## 7. Urethral Sphincter BoNT-A Injection in Dysfunctional Voiding

As urethral BoNT-A injection had been successfully used in the treatment of DSD in SCI patients, this treatment was further applied to adults with non-neurogenic voiding dysfunction due to bladder outlet obstruction and urethral sphincter overactivity. Fowler’s syndrome consists of difficulty in passing urine or urinary retention due to failure to relax the urethral sphincter in patients without neurological or anatomical abnormality [46]. Treatment of Fowler’s syndrome is complicated and patients usually need intermittent self-catheterization [46]. In 2016, an open-label, prospective study enrolled 10 women with difficult urination due to Fowler’s syndrome and treated these patients with urethral injection of 100 U of BoNT-A [47]. At 10 weeks of follow-up, the maximal urinary flow rate was significantly increased and the residual volume was decreased. Even four of the five women, who initially had complete retention, could void spontaneously after the treatment. Recently, we also conducted a randomized, double-blind, and placebo-controlled study using BoNT-A injection into the urethral sphincter to treat patients with refractory DV (open bladder neck but a poorly relaxed urethral sphincter, and a normal-to-high voiding pressure with a low urinary flow) [48]. Our results revealed that patients who received BoNT-A injection had a significantly improved international prostate symptom score (IPSS), quality-of-life index, maximum flow rate, voided volume, and decreased detrusor voiding pressure at one month of follow-up. When compared with the normal saline injection group, however, only the total IPSS and voided volume improvement were significantly greater in the BoNT-A injection group. Improvement in other clinical parameters was not significantly different between the BoNT-A and normal saline injection groups. We concluded that urethral sphincter injection with either BoNT-A or placebo could safely and effectively ameliorate clinical symptoms and improve quality of life in patients with DV. Although the exact pathogenetic mechanism remains unknown, local injection itself might have a therapeutic effect on the relaxation of the urethral sphincter, regardless of pharmacologic effects of BoNT-A. Additional studies enrolling more patients with DV are necessary to elucidate the efficacy of BoNT-A urethral injection.

## 8. Intraprostatic BoNT-A Injection in Benign Prostate Hyperplasia

Benign prostate hyperplasia (BPH) resulting in bladder outlet obstruction is one of the most common conditions presented in the urology clinic. An epidemiological meta-analysis revealed the occurrence of BPH in age groups 40–49 years, 50–59 years, 60–69 years, 70–79 years, and 80 years and older was 2.9%, 29.0%, 44.7%, 58.1%, and 69.2%, respectively [49]. Mainstream treatment of BPH is usually started with oral medication including an α-adrenergic blocker and a 5-α-reductase inhibitor [50]. Surgical intervention such as transurethral prostate resection is indicated if BPH patients are refractory to treatment with oral medication [51]. The prostate is an organ composed of glandular tissue and fibromuscular stroma. The prostate may cause bladder outlet obstruction not only because of glandular hyperplasia, but also because it may be associated with the dysregulation of smooth muscle contractility in the stroma [52]. Relaxation of smooth muscle in the prostate stroma has been considered a potential target to treat patients with BPH in many pharmacological studies [52]. Given its inhibitory effect on neurotransmitter release from the neuromuscular junction, studies using 100 to 200 U intraprostatic injection of BoNT-A to treat patients with BPH started as early as 2003, and initial results showed promising therapeutic effects [53,54]. BPH patients who received intraprostatic BoNT-A injection experienced significant improvement in both symptom score and quality-of-life index at short-term follow-up (one month) [53,54]. In addition, evidence from both human and animal studies showed prostate apoptosis activity increased after BoNT-A injection [53,55]. However, two large, randomized, double-blind, placebo-controlled trials showed no significant difference between patients who received intraprostatic BoNT-A injection and placebo [56,57]. A meta-analysis including randomized, placebo-controlled trials also suggested that BoNT-A injection for patients with BPH does not have a significantly better therapeutic effect than does placebo [58]. On the other hand, a recent randomized placebo-controlled study, which enrolled only BPH patients with moderate to severe symptoms (IPSS ≥ 19) and pressure-flow study, indicated bladder outlet obstruction showed different results [59]. Patients received BoNT-A injection reported significantly greater improvement of IPSS, maximum flow rate, and PVR when compared with the placebo group at three months of follow-up. The follow-up urodynamic study in the BoNT-A injection group also showed a significant reduction in bladder outlet obstruction index (54%), which was not significantly changed in the placebo group. This study enrolled only BPH patients with evidence of bladder outlet obstruction and might be the reason why these results are different from previous studies. We suggested intraprostatic BoNT-A should be a reasonable treatment option for moderate to severe BPH patients who are refractory to oral medications and not willing to undergo surgical intervention. Careful selection of patients according to symptom severity and urodynamic study evaluation before treatment might be essential for successful outcome of this treatment.

## 9. Intraprostatic BoNT-A Injection in Chronic Prostatitis

Prostatitis is another common problem among many young and middle-aged male patients in the urology clinic. Male patients with chronic prostatitis usually present with pelvic pain/discomfort (perineal, testicular, penis, or pubic area) and voiding symptoms [60]. Treatments of chronic prostatitis should be treated first with oral antibiotics and an α-adrenergic blocker [61]. If there is no obvious symptomatic benefit, medications targeting neuropathic pain or neuromodulation procedures should be considered in these patients [61]. Giorgio et al. first used BoNT-A injection to treat voiding dysfunction in male patients with chronic prostatitis [62]. An animal study revealed the anti-inflammatory and analgesic effects of BoNT-A in prostatitis [63]. In rats with capsaicin-induced prostatitis, Chuang et al. showed that intraprostatic BoNT-A injection could both reduce pain and decrease infiltration of inflammatory cells in the prostate. Falahatkar et al. conducted a prospective, randomized, double-blind, placebo-controlled study to evaluate transurethral intraprostatic injection of 100 U of BoNT-A for patients with chronic prostatitis [64]. Significant improvement in pain score, National Institutes of Health chronic prostatitis symptom index, and quality of life were observed in the BoNT-A injection group at six months of follow-up. When compared with the placebo group, symptom improvements were significantly greater in the BoNT-A group. Another study compared the efficacy of transurethral and transrectal intraprostatic BoNT-A for chronic prostatitis [65]. Both groups demonstrated significant improvement in pain at six months of follow-up, but only patients in the transrectal injection group had significant improvement in the chronic prostatitis symptom index. Both anti-inflammatory and anti-nociceptive effects should be key factors for treating chronic prostatitis with BoNT-A. Although these pilot studies showed promising results of BoNT-A injection for treating chronic prostatitis, additional randomized placebo-controlled trials that enroll more patients are necessary to prove its efficacy.

In summary, both researchers and clinicians are determined to develop novel BoNT-A applications for treating LUTDs, improve the convenience of drug delivery, and decrease adverse effects. However, some patients continued to be dissatisfied with the outcome and opted to discontinue injections. For example, in a prospective study, only 68% of refractory OAB patients would like to continue to receive BoNT-A treatment after the first injection [66]. A study also detected neutralizing antibodies to BoNT-A in patients who had received bladder or urethral BoNT-A injections and suggested a possible cause of therapy failure [67]. Physicians should comprehensively evaluate voiding problems in patients and make diagnoses precisely before using BoNT-A to treat LUTDs. Further research that focuses on ways to improve BoNT-A with stronger effects and long-lasting formulations are necessary. Adjustment of BoNT-A dose according to urodynamics, study findings, or in combination with oral medications might improve the efficacy or prolong the duration of the therapeutic effect. A summary of novel applications of BoNT-A in LUTDs is provided in Table 1.

## 10. Conclusions

Since BoNT-A was first used to treat LUTD 30 years ago, applications have become increasingly prevalent and popular. Intravesical BoNT-A injection for patients with OAB or NDO has been widely used in daily urologic practice and proved by DHIC in the United States and many countries. For IC/BPS patients who have an inadequate response to initial treatment, intravesical BoNT-A injections also have been considered as standard treatment according to different clinical guidelines. BoNT-A delivery with liposome-encapsulation and gelation hydrogel intravesical installation provided a new class of less invasive and convenient application for patients with OAB or IC/BPS. Clinical trials revealed promising therapeutic results of novel BoNT-A applications, including DV, BPH, and chronic prostatitis. However, further randomized and placebo-controlled studies that enroll patients with accurate diagnoses are necessary to prove the efficacy of BoNT-A treatment. Both careful patient selection and prudent use of urodynamic study evaluation to confirm diagnoses are essential to achieve successful treatment outcomes.

## Figures and Tables

**Table 1:** Summary of novel applications of BoNT-A in Lower urinary tract dysfunctions (LUTDs).

LUTDs	Condition	BoNT-A Delivery Route	Study Design	Efficacy	Comment
LUTDs originated from bladder	IC/BPS	Intravesical injection	Randomized, placebo-controlled	Significantly greater reduction in pain and increase in bladder capacity in BoNT-A group compared with placebo group	First randomized, placebo-controlled trial for IC/BPS
		Instillation Lipotoxin	Randomized, placebo-controlled	No significant difference between Lipotoxin and placebo group	-
		Instillation gelation hydrogel	Prospective, non-controlled study	BoNT-A mixed with hydrogel significantly reduced the pain score at 12 weeks of follow up	-
	OAB	Instillation Lipotoxin	Randomized, placebo-controlled	Significantly improved frequency and urgency in Lipotoxin group but not in placebo group	-
	DHIC	Intravesical injection	Retrospective study	Subjective urgency symptom score significantly improved, but not incontinence episode	33% of patients experienced retention
LUTDs originated from bladder outlet	DV	Intrasphincter injection	Randomized, placebo-controlled	Significantly improved QoL, Qmax, IPSS, and VV in the study group. Only IPSS and VV improved greater than placebo group	-
	BPH	Intraprostatic injection	Randomized, placebo-controlled	Significantly greater improvement in IPSS, Qmax, and PVR compared with placebo group	Select patients with BPH urodynamic study
	Chronic prostatitis		Randomized, placebo-controlled	Significant improvement in pain score and QoL compared with placebo group	-

QoL: quality of life; Qmax: maximal urinary flow rate; IPSS: international prostate symptom score; VV: voided volume; PVR: post-voiding residual volume. IC/BPS: interstitial cystitis/bladder pain syndrome; OAB: overactive bladder; DHIC: detrusor hyperactivity with impaired contractile function; DV: dysfunctional voiding; BPH: benign prostate hyperplasia.
